# Improving accuracy of opening-wedge osteotomies of distal radius using a patient-specific ramp-guide technique

**DOI:** 10.1186/s12891-018-2279-0

**Published:** 2018-10-15

**Authors:** Simon Roner, Fabio Carrillo, Lazaros Vlachopoulos, Andreas Schweizer, Ladislav Nagy, Philipp Fuernstahl

**Affiliations:** 1 Computer Assisted Research and Development Group, Balgrist University Hospital, University of Zurich, Forchstrasse 340, 8008 Zurich, Switzerland; 2 Department of Orthopaedics, Balgrist University Hospital, University of Zurich, Zurich, Switzerland

**Keywords:** Osteotomy, Distal radius, Extra-articular, Patient specific-instruments, Computer-assisted planning, Ramp-guide, Malunion

## Abstract

**Background:**

Opening-wedge osteotomies of the distal radius, performed with three-dimensional printed patient-specific instruments, are a promising technique for accurate correction of malunions. Nevertheless, reports of residual malalignments and discrepancies in the plate and screw position from the planned fixation exist. Consequently, we developed a patient-specific ramp-guide technique, combining navigation of plate positioning, osteotomy cutting, and reduction. The aim of this study is to compare the accuracy of navigation of three-dimensional planned opening-wedge osteotomies, using a ramp-guide, over state-of-the-art guide techniques relying solely on pre-drilled holes.

**Methods:**

A retrospective analysis was carried out on opening-wedge osteotomies of the distal radius, performed between May 2016 and April 2017, with patient-specific instruments. Eight patients were identified in which a ramp-guide for the distal plate fixation was used. We compared the reduction accuracy with a control group of seven patients, where the reduction was performed with pre-drilled screw holes placed with the patient-specific instruments. The navigation accuracy was assessed by comparing the preoperative plans with the postoperative segmented, computed tomography scans. The accuracy was expressed using a 3D angle and in measurements of all six degrees of freedom (3 translations, 3 rotations), with respect to an anatomical coordinate system.

**Results:**

The duration of the surgery of the ramp-guide group was significantly shorter compared to the control group. Significantly less rotational and translational residual malalignment error was observed in the open-wedged osteotomies, where patient-specific instruments with ramp-guides were used. On average, a residual rotational malalignment error of 2.0° (± 2.2°) and a translational malalignment error of 0.6 mm (± 0.2 mm) was observed in the ramp-guide group, as compared to the 4.2° (± 15.0°) and 1.0 mm (± 0.4 mm) error in the control group. The used plate was not significantly positioned more accurately, but significantly fewer screws (15.6%) were misaligned in the distal fragment compared to the control group (51.9%).

**Conclusion:**

The use of the presented ramp-guide technique in opening-wedge osteotomies is improving reduction accuracy, screw position, and surgical duration, compared to the existing patient-specific instrument based navigation methods.

## Background

Malunions are one of the most common complications following fractures of the distal radius. As a consequence of alterations in the alignment, patients may have a limited range of motion (ROM) in the wrist and forearm or a painful or unstable distal radioulnar joint [1]. Typically, malunions of the distal radius fractures include a shortening and, especially after a Colles’ type fracture, a dorsal angulation of the distal fragment [2]. Therefore, malunited distal radius fractures are often corrected through opening-wedge osteotomies.

Several studies have demonstrated the accurate restoration of radiological indices using corrective opening-wedge osteotomies to relieve the pain, and to improve the range of motion in symptomatic patients [3]. Nevertheless, three-dimensional (3D) deformities, especially with rotational malalignment, are not assessed accurately in two-dimensional (2D) plain radiographs [4]. A more reliable method is using 3D bone models, extracted from computed tomography (CT) for a precise deformity analysis, and preoperative planning for the corrective osteotomy [5–7]. Patient-specific instruments (PSI) for navigation can be designed based on the 3D-simulated postoperative bone models to accurately execute the surgical plan. A study in 2015 by Vlachopoulos [8] demonstrated a high reduction in the accuracy of closing-wedge and single-cut osteotomies, with a residual rotational malalignment error of 3.5° (± 1.1°), when using patient-specific instruments. Opening-wedge osteotomies were significantly less accurate at 8.30° (± 5.35°). The authors mention high soft-tissue tension as one of the reasons for difficulties when performing the reduction of opening-wedge osteotomies with only pre-drilled screw holes as reference.

Due to the weakness of the current PSI systems, we have developed a new patient-specific guide technique, for optimal plate and screw positioning in opening-wedge osteotomies. In 2016, the method was presented as a case of corrective osteotomy of a complex, malunited humeral fracture. With the aid of a ramp-guide, the reduction was performed indirectly using the predefined fixation of the plate on the humeral head [9]. The main feature is to create a guide for the navigation of the plate position using a ramp-like surface, on which the implant can be mounted, hereinafter called a ramp-guide. The goal of this study is to retrospectively compare the accuracy of the 3D planned opening-wedge osteotomies of the distal radius, using a ramp-guide for distal plate fixation, to that of the state-of-the-art patient-specific guides.

## Methods

Nine patients were identified who underwent osteotomies of the distal radius, using patient-specific ramp-guides, between May 2016 and April 2017. The criteria for inclusion were the performance of an extra-articular opening-wedge osteotomy, and the presence of a postoperative CT scan. The control group was operated on between January and September 2013 in our department and consisted of seven patients who had been treated with open-wedge osteotomies, using commercial, state-of-the-art, patient-specific instruments (MyOsteotomy; Medacta SA, Castel San Pietro, Switzerland). The reduction accuracy of the control group was published in 2015 by Vlachopoulos [8]. Demographics of both groups are listed in Table 1.

**Table 1:** Demographic data, clinical data, and type of corrective osteotomy is given to each patient

Group	Patient	Age ranges (years)	Side	Injury to surgery (months)	Previous treatment
Ramp Guide	1	60–69	right	12	cast
	2	50–59	right	7	cast
	3	40–49	left	274	cast following surgery
	4	50–59	left	7	cast
	5	50–59	left	11	cast
	6	40–49	left	218	surgery
	7	50–59	right	10	cast
	8	60–69	right	14	cast
Control	9	30–35	right	9	cast
	10	10–19	left	33	cast
	11	10–19	left	9	cast
	12	10–19	right	40	cast
	13	10–19	left	no trauma	none
	14	10–19	left	5	surgery
	15	40–49	right	13	cast

### Preoperative planning

Segmentations of pathological and contralateral healthy bone models from the CT data (Philips Brilliance 40 T, Amsterdam, Netherlands) were generated using commercial software, as discussed in a previous publication [8]. In-house preoperative planning software (CASPA; Balgrist CARD AG, Zurich, Switzerland) was used to create the 3D preoperative surgical plan. The mirror model of the contralateral bone was used as a template representing the physiological anatomy. The basic principle of 3D planning of opening-wedge osteotomies has been described previously [8].

### Guide design

**Fig. 1:**
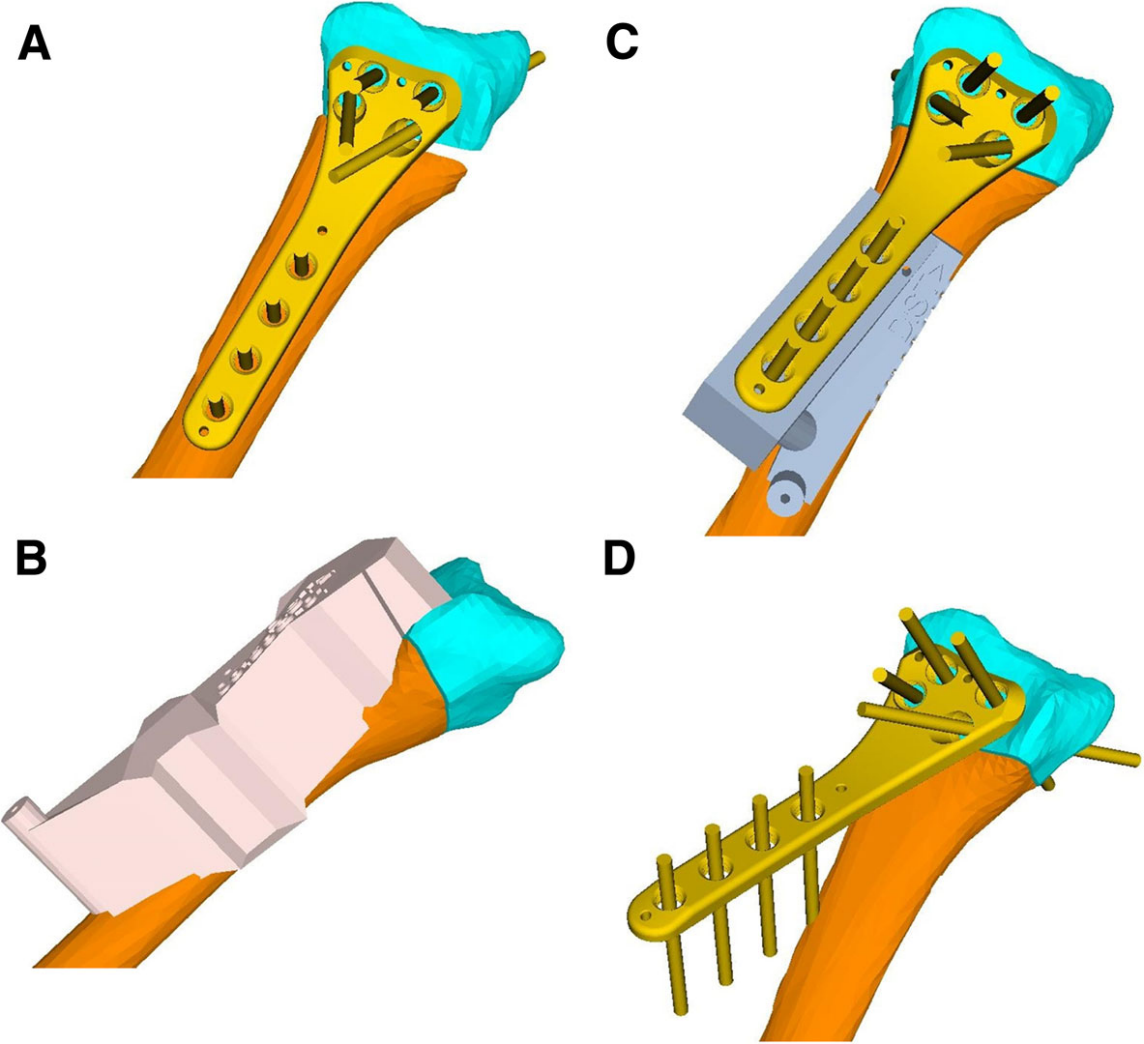
**a** Planned opening-wedge osteotomy with shaft (orange) and distal fragment (cyan) with optimal plate position (yellow). **b** Pre-reduction guide (pink) with optimal fit on the malunited distal radius with cutting slit. **c** Ramp guide (gray) attached to malunited distal radius with fixated plate (yellow) on the slope. **d** Fixated plate (yellow) on the distal fragment before completing the osteotomy

The plate is positioned exactly as desired on the simulated postoperative result (Fig. 1a). The position and direction of the screws in the proximal fragment are used to first design a pre-reduction guide on the surface of the pathological bone (Fig. 1b), used to pre-drill the proximal screws into the shaft fragment. This guide is also comprised of a cutting slot for partially navigating the osteotomy. In a second step, the plate is back-transformed on the pathological bone by applying the reduction to the plate and the distal fragment (Fig. 1c). Note that the position of the distal plate part relative to the distal fragment, did not change. Therefore, the back-transformation defines how the plate must be fixed on the distal fragment, before completing the corrective osteotomy. The ramp-guide is designed upon the pathological bone (Fig. 1d), defining the position of the plate, with respect to the distal fragment. Accuracy is guaranteed by a negative footprint of the plate on the slope, with a hole for the possibility of temporarily fixating the planned plate with a screw.

Patient-specific instruments of the control group were designed, as published in 2015 by Vlachopoulos [8]. In general, pre-reduction guides were used to define the proximal and distal holes of the plate and the position of the cutting plane. A senior surgeon (A.S or L.N.) verified the 3D preoperative plan for both groups. Consequently, the patient-specific guides were designed by the same in-house planning software and produced externally by Medacta SA (Castel San Pietro, Switzerland).

### Surgical technique

**Fig. 2:**
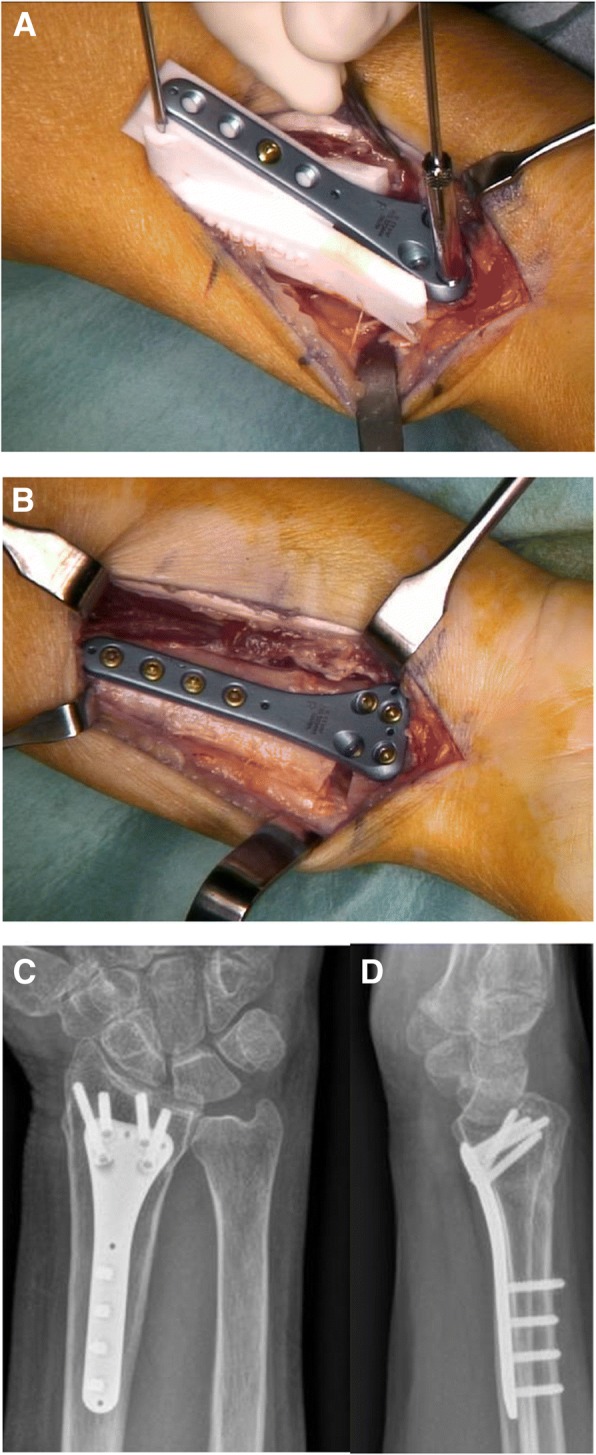
**a** Ramp-guide (white) with temporal fixation by K-wires on the most proximal drilling-hole. **b** Plate reduction with fixated distal fragment after guide removal. **c** and **d** Posteroanterior and lateral radiographs after corrective osteotomy

A standard modified Henry approach was performed to expose the malunion of the distal radius. After careful surface preparation of the malunited bone, the pre-reduction guide was applied, with K-wires defining the proximal screw hole positions. Subsequently, less than the half of planned osteotomy was performed without losing the connection and position of the distal fragment. Optionally, this step of partially navigate the planned osteotomy can be included in the ramp-guide itself. After removing the pre-reduction guide, the described ramp-guide was fixed proximally, using the same K-wires to guarantee accurate guide position. The plate was latched on the ramp in the negative footprint and temporarily fixated with a cortical screw. Subsequently, drilling using conventional angular-stable sleeves (Intercus AG, Aarau, Switzerland), the plate was attached to the distal fragment with locking screws (Fig. 2a). To complete the osteotomy, the ramp-guide was detached from the plate after losing the ramp-screw and removal in the proximal direction. The final step is performing the reduction of the plate, with the fixated distal fragment in the pre-defined proximal screw holes on the shaft **(**Fig. 2b**)**. After a fluoroscopy verification of the reduction and the screw lengths, a volar splint was applied on the wrist post wound closure. A two-week postoperative, clinical control was scheduled with suture removal. Aftercare was conducted with immobilization in a splint, and with functional treatment provided by a hand therapist for the first eight weeks.

Similar to the ramp-guide technique, pre-reduction guides were used to position and perform the osteotomy in the control group. After removing the guide, the planned plate involving locking screws was fixated on the distal fragment using the predefined direction of the drilled screw holes. Subsequently, reduction of the plate with the fixed distal fragment, as previously described was performed.

### Evaluation method

**Fig. 3:**
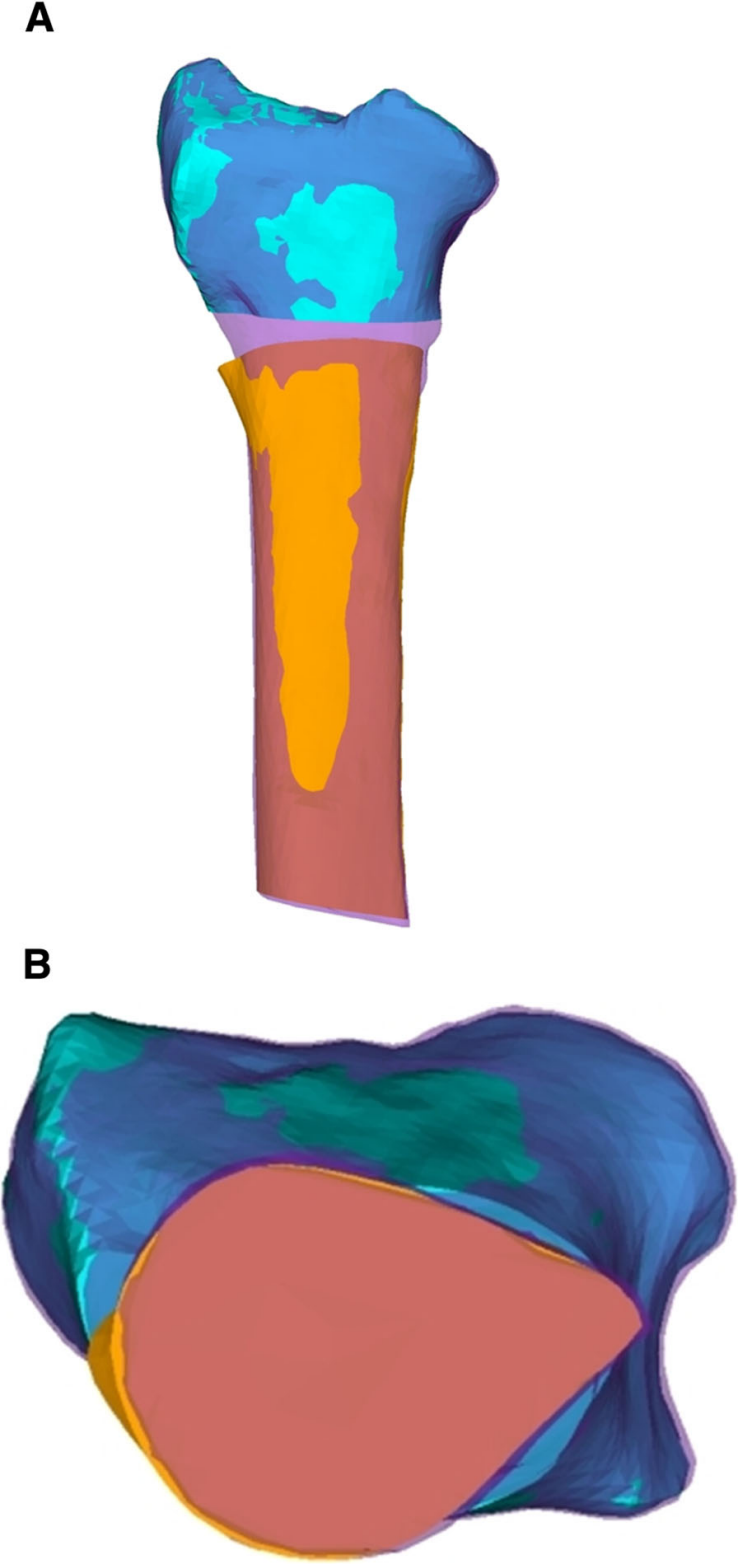
**a** and **b** A Case with superimposition of the postoperative result in transparency mode (purple) with the surgical plan (orange and magenta). This demonstrates the alignment of the corticalis (**b**) when only the distal part of the radius is available

**Fig. 4:**
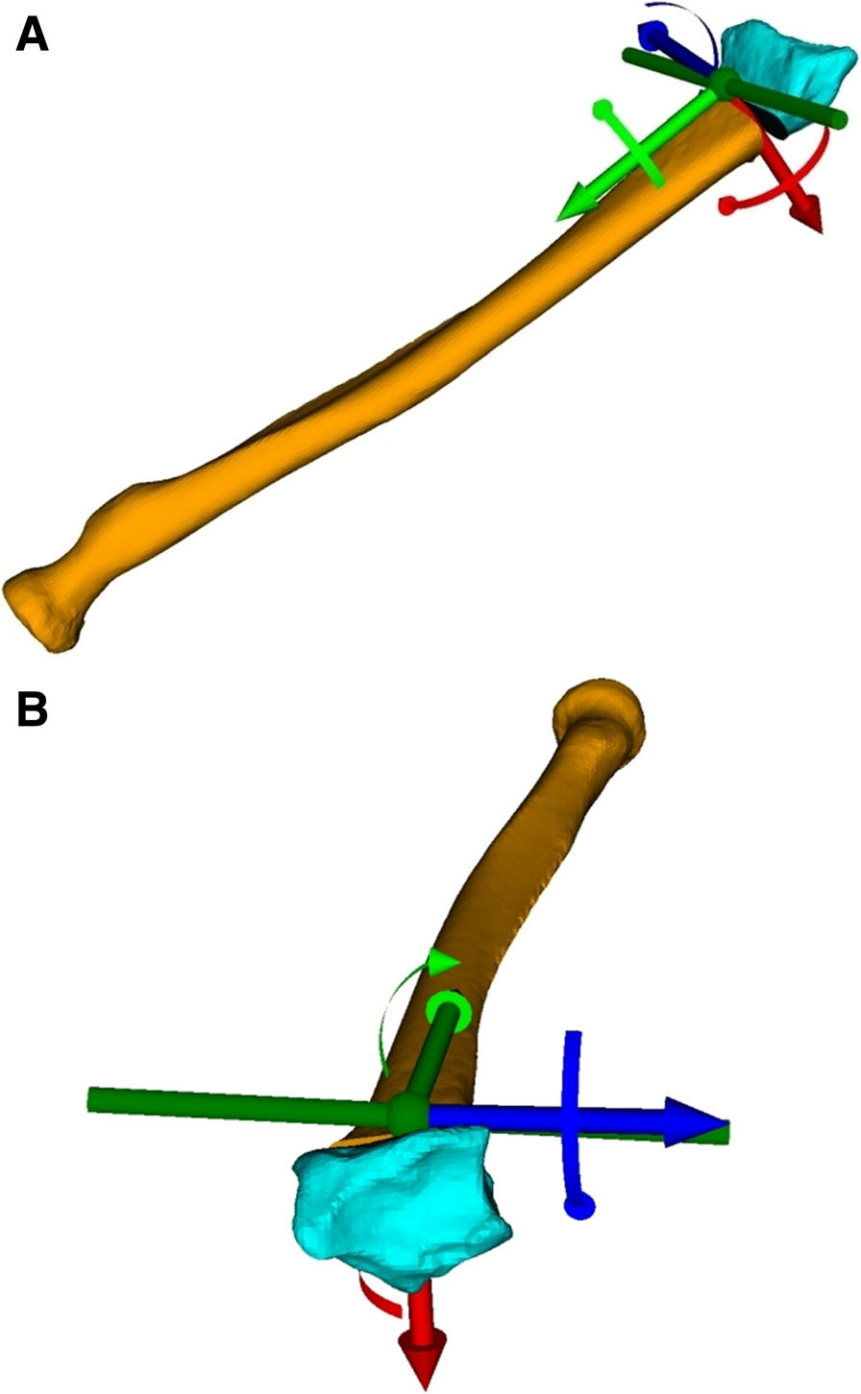
**a** and **b** Center of the coordinate system, defined on the rotation axis (dark green) of the fragment transformation

After eight weeks of postoperative care, a CT scan of the operated forearm was acquired to verify consolidation. As described in a previous publication [8], the residual malalignment error was measured by comparing the planned model with the postoperative result in 3D. Additionally, the postoperative plate and screw position was compared to the position in the 3D planning. Only if the distal part of the postoperative radius was present in the postoperative CT scan, axial alignment of the postoperative bone model with the model of the malunion was performed as follows: After superimposing, using the Iterative Closest Point (ICP) surface registration algorithm [7], the exact visual alignment of the cortical cross-section, on the proximal end of both models, was conducted by displaying the postoperative bone model in transparency mode (Fig. 3). The residual malalignment error was measured by calculating the relative transformation between the distal radius parts in planned and postoperative positions. The resulting 4 × 4 transformation matrix was decomposed in a rotational and translational part. The residual angulation was expressed with an axis-angle representation; additionally, as three consecutive rotations (i.e., Euler rotations) [10] around a standardized coordinate system, as depicted in Fig. 4. An anatomic coordinate system was set, with the x-axis pointing from dorsal to volar, defining the ulnar−/radial rotation axis. The y-axis was defined as perpendicular to the x-axis, pointing in the direction of the anatomical axis of the radius in the proximal direction. The z-axis was defined to be orthogonal to the x- and y-axes in the ulnar direction.

### Statistical analysis

After the graphical verification of normal distribution, an unpaired t-test was performed to compare the residual rotational and translational error in absolute values, separately between both groups; the level of significance was established at *p* < 0.05. Likewise, the residual error of the postoperative plate position was compared to the plan, using an unpaired t-test between both groups. Comparison of age and operation time between the two groups was conducted using the Mann-Whitney U Test; the level of significance was established at p < 0.05. The software R (Version 3.4.3; R Foundation, Vienna, Austria) was used for statistical evaluation.

## Results

The demographic data is summarized in Table 1**.** The ramp-guide group (mean age 42; range 30–66) was significantly older, compared to the control group (mean age 15; range 12–45). Consolidation of the osteotomy was observed in all patients between weeks 8 and 10 on the CT scans. No postoperative complications were reported in either of the two groups. In all ramp-guide cases, a correctus plate (Intercus AG, Aarau, Switzerland) was used; an implant with an uniaxial locking screw system. In the control group, angular-stable plate systems from two different companies (Intercus AG, Aarau, Switzerland; Synthes, West Chester, USA) were used.

**Fig. 5:**
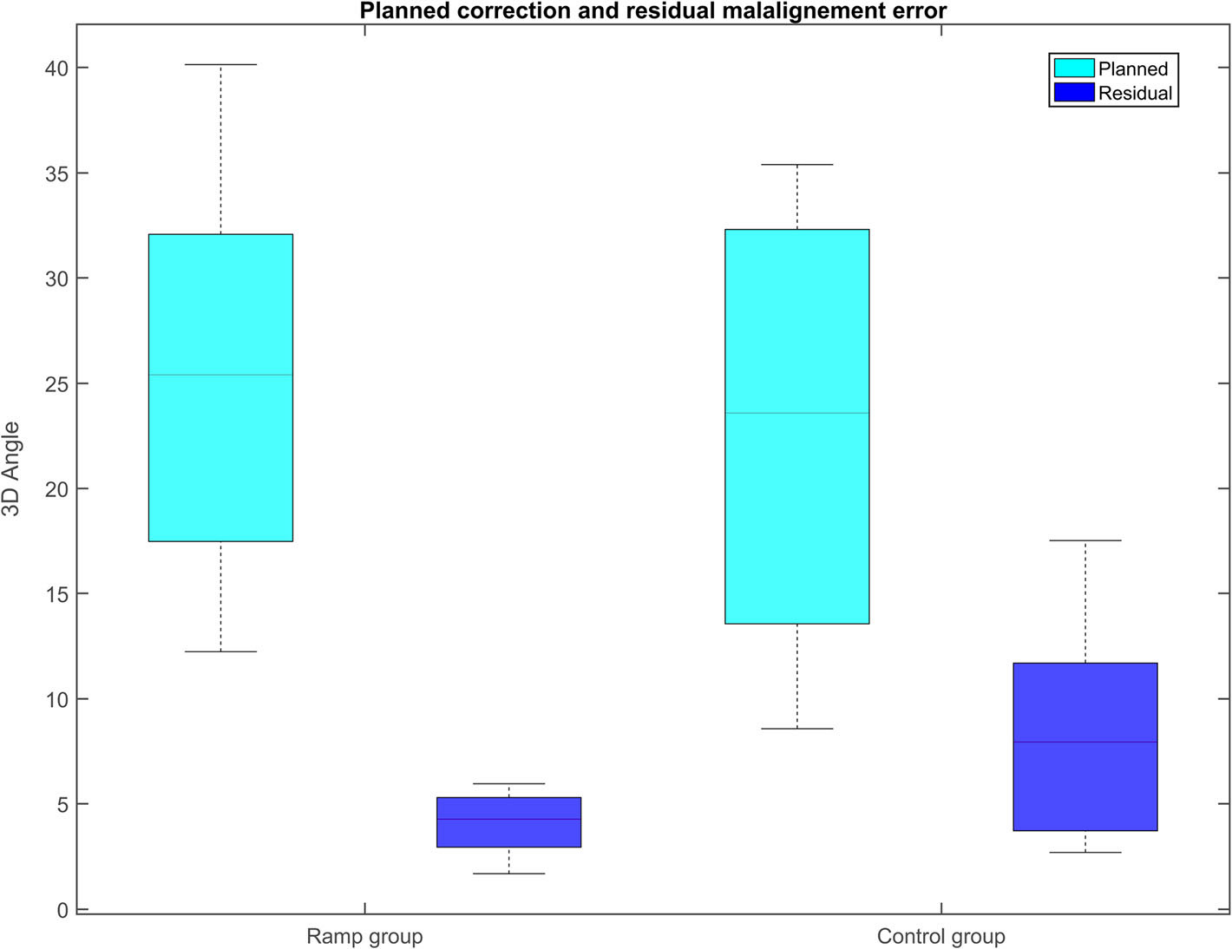
Planned correction and residual malalignment error of both groups expressed as 3D angle

**Fig. 6:**
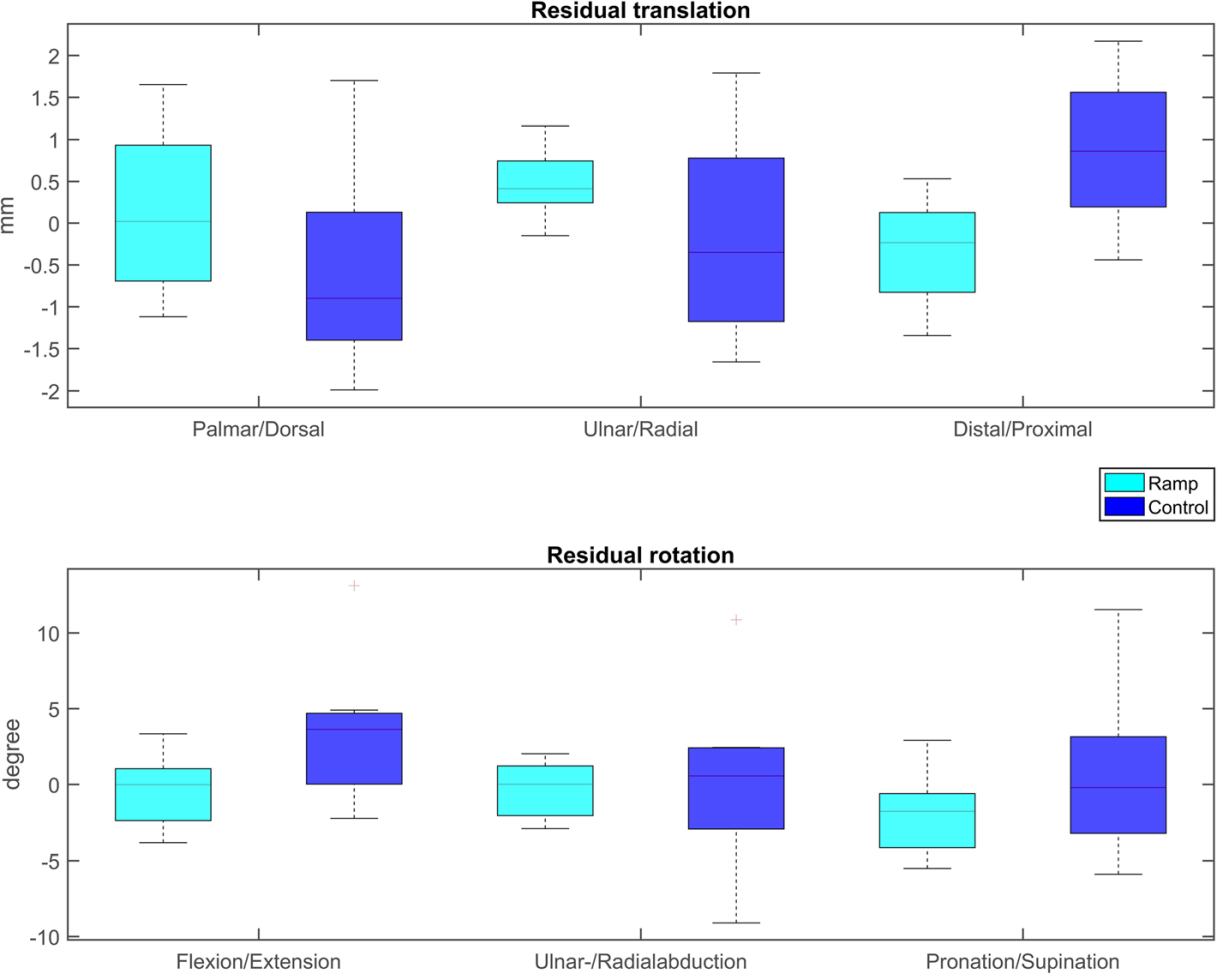
Residual malalignment error measured in all six degrees of freedom (3 translations, 3 rotations) with respect to the anatomical coordinate system

**Fig. 7:**
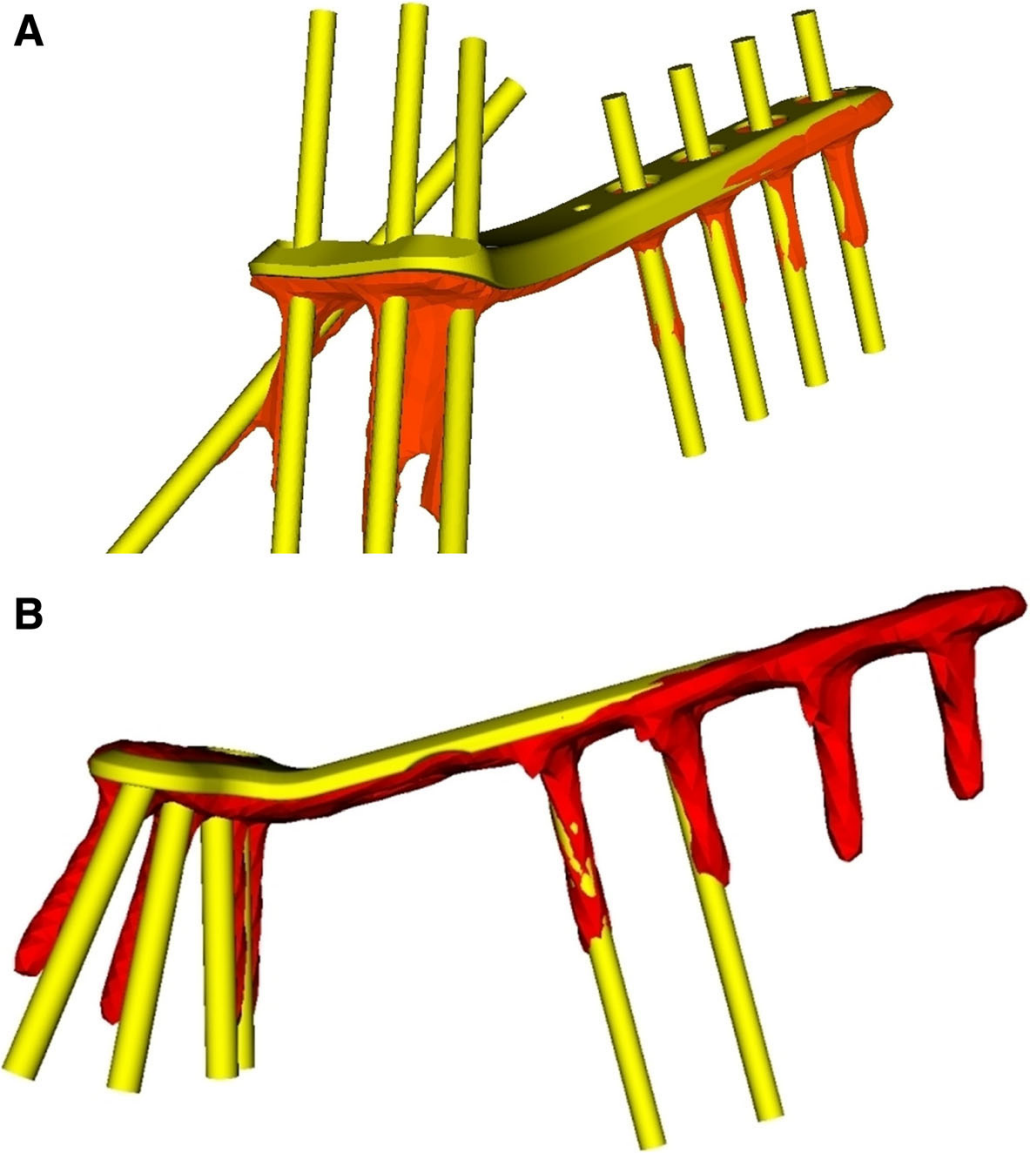
Example of plate and screws of 3D planned (yellow) and obtained (orange) position of each group. **a** A case of the ramp-guide group with corresponding screws directions. **b** A case of the control group with mismatch of screw directions

The measurements of the planned correction and the residual malalignment error, with a calculated 3D axis-angle around the coordinate system, are summarized in Fig. 5. The interventional group showed a significantly lower residual rotational error of 2.0° (± 2.2°), and translational error of 0.6 mm (± 0.2 mm) than the control group, where the rotational and translation error were 4.2° (± 15.0°) and 1.0 mm (± 0.4 mm), respectively (Fig. 6). Instances of plate and screw position of each group are depicted in Fig. 7**.** Compared to the planned plate position, a mean, residual rotational error of 1.3° (± 2.3°), and a translational error of 0.6 mm (± 0.2 mm) of the plate was measured in the ramp-guide group. No significant differences in the residual plate error was observed compared to the control group, where a rotational error of 2.0° (± 5.8°) and translational error of 0.7 mm (± 0.2 mm) was measured. Significantly, in the ramp-guide group, 5 of the total 32 (15.6%) screws in the distal fragment were misaligned (> 10°), compared to the 14 of a total of 27 (51.9%) in the control group. The duration of the surgery for the ramp-guide group (86–161 min) was significantly shorter compared to the control group (140–240 min).

## Discussion

Performing open-wedge osteotomies planned in 3D, and using patient-specific instruments, is a promising method to accurately restore bone anatomy of malunited distal radius. Several navigation techniques have been described to precisely achieve the desired reduction after performing the osteotomy. In laboratory studies, comparable results were reported between K-wire based specific reduction guides [11], and patient-specific drill guides with reduction based on the pre-drilled screw directions [12]. In 2013, Kunz et al. performed distal radius osteotomies on nine patients, with patient-specific drill guides based on the implant screw directions [13]. Similarly, Vlachopolous et al. reported a high reduction accuracy in 3D-planned closing-wedge and single-cut osteotomies of the forearm, performed with PSI [8]. A significantly higher residual rotational error was observed in opening-wedge osteotomies (8.30° ± 5.35°). In two cases a residual deformity of above 10° was reported. Excessive lengthening of the bone, additional to the rotational component, was required in both cases to achieve the planned reduction, resulting in very high soft tissue tensioning. They reported difficulties performing the reduction, solely based on the pre-drilled screw holes, and suggested that the stress between the fragments might additionally cause the screws to lock into the plate somewhat differently from the planned direction. Miyake et al. [14] performed opening-wedge osteotomies of the distal radius on 10 patients, using a patient-specific guide technique, with prior plate-fixation to the distal radius fragment [15]. In contrast to the hereby described ramp-guide technique, the plate was fixated freehand by pre-drilling the screw holes on the distal fragment. The average deviation between the achieved and planned radial inclination was 1.8° (± 0.8°), for volar tilt, the average deviation between planned and achieved angle was 1.9° (± 1.5°).

In this study, we compared the accuracy of a new guide technique, where additionally the plate positioning was navigated with a patient-specific ramp-guide. Compared to state-of-the-art PSI techniques, using the pre-drilled screw holes, the hereby presented ramp-guide technique facilitates plate positioning on the distal fragment, and ensures a more accurate placement of the screws in the desired direction. Performing the reduction of the plate on the radius shaft, with a fixed and stable distal fragment, is technically less demanding in our opinion. Plate positioning on the distal radius prior to osteotomizing the radius can be performed more accurately, and therefore reduce misalignments. Postoperative 3D evaluation confirms this assumption by demonstrating a higher reduction accuracy in the ramp-guide group, compared to the control group. Rotational and translational residual malalignment error was significantly less, in each of the anatomical planes, in the ramp-guide group.

Comparison of the plate position between a 3D planned and performed surgery, did not support our assumption of significantly better implant positioning. Tough no outliners were detected in the ramp-guide group, contrasted by two outliners in the control group. Furthermore, in the control group 14 out of 27 (51.9%) of the screws in the distal fragment were misaligned compared to 5 out of 32 (15.6%) in the ramp-guide group. However, accurate screw placement is still an issue in corrective osteotomies, as incorrectly placed screws can compromise the planned reduction. In our opinion a larger thread on the plate, as used in the ramp-guide group, can support the correct direction of screws. Further investigation should be conducted on how to navigate screw placement more precisely, including the verification of cannulated screw application.

Despite the promising results and shorter surgery duration, the technique is only suitable for a subset of malunions. Application of a ramp-guide from palmar is only possible for malunions needing correction towards a palmar direction. The application of a ramp-guide from the opposite side may be an option to overcome this limitation. Application areas for a ramp-guide technique could be extended in the future, particularly in corrections desiring high accuracies. Further investigations are required to address PSI expansions in various anatomical regions.

Drawbacks of this technique could be the more challenging and time-consuming design of the ramp-guide, compared to state-of-the-art PSI. Furthermore, by using a ramp-guide the partially performed osteotomy must be completed freehand. The weakness of this study is the retrospective character and the small sample size. No clinical outcome was reported, since the aim of this study was to analyze the accuracy of different guide techniques. Nevertheless, the above mentioned studies demonstrated good clinical outcome in accurate restoration of forearm malunions, with PSI, without disadvantages. Finally, comparison of the outcome, with a long-standing follow-up of different techniques and accuracy results is necessary.

## Conclusion

This study demonstrates the further development of patient-specific instruments in performing corrective osteotomies. The use of the presented ramp-guide technique in opening-wedge osteotomies of the distal radius is improving accuracy and reducing surgery duration, compared to existing state-of-the-art methods using PSI. Moreover, screw positioning is more accurate with the guide-ramp design.

## Appendix

**Table 2:** Preoperatively planned correction and residual malalignment error after surgery are given for each patient

Group		Planned Correction							Residual error						
		Rotation (°)				Translation (mm)			Rotation (°)				Translation (mm)		
	Patient	3D angle	Flexion / Extension	Ulnar / Radial	Pronation Supination	Palmar / Dorsal	Ulnar / Radial	Distal / Proximal	3D angle	Flexion / Extension	Ulnar / Radial	Pronation Supination	Palmar / Dorsal	Ulnar / Radial	Distal / Proximal
Ramp guide	1	26.82	26.58	3.15	−2.63	0.00	−0.05	0.01	4.45	3.34	−2.86	−0.61	0.25	0.77	−0.18
	2	27.83	19.93	10.02	−18.63	0.47	−1.29	1.43	5.71	1.01	1.12	−5.52	1.65	0.27	0.06
	3	14.64	5.35	3.74	−12.94	0.95	1.26	2.80	2.24	−1.21	0.09	−1.99	1.28	0.31	−0.58
	4	12.24	11.28	2.71	−4.20	− 0.06	0.29	− 0.12	5.95	−3.84	− 0.06	− 4.55	−0.95	0.72	−1.35
	5	23.98	23.57	3.14	−3.91	2.58	−2.07	−8.74	4.88	1.06	−2.92	−3.80	−0.21	0.22	0.19
	6	36.31	34.33	4.49	−12.62	0.00	−0.26	0.11	1.69	−0.89	1.31	−0.61	−0.44	− 0.15	−0.29
	7	20.29	19.55	4.94	−3.33	0.45	2.03	−0.43	4.08	−3.57	−1.26	−1.56	−1.12	0.51	−1.08
	8	40.13	40.03	−2.54	−0.93	− 0.80	1.15	0.00	3.66	0.85	2.02	2.91	0.58	1.16	0.53
Control	9	22.79	19.33	10.98	−7.39	−1.66	1.16	5.31	7.94	4.90	2.33	−5.90	0.10	1.79	0.86
	10	10.49	5.78	8.76	−0.71	0.55	0.36	7.66	4.76	3.63	0.56	−3.05	−1.99	−0.31	1.60
	11	28.26	24.34	−9.81	12.96	−0.54	0.81	9.08	17.51	13.08	−3.74	11.50	−0.92	−1.66	1.44
	12	35.39	7.42	32.61	9.84	0.09	1.15	17.93	12.47	4.06	10.86	4.25	−1.56	−1.21	2.17
	13	23.58	−10.46	−19.88	5.70	0.09	0.72	5.81	9.38	−2.24	−9.11	−0.21	1.70	1.14	−0.44
	14	33.65	−12.59	−16.12	−28.73	1.13	1.50	1.93	3.36	−0.36	− 0.55	−3.29	−0.90	−1.08	0.08
	15	8.57	−2.51	0.48	8.19	4.43	4.57	1.83	2.70	1.14	2.44	−0.18	0.14	−0.35	0.53

## Abbreviations

2D: Two-dimensional
3D: Three-dimensional
CT: Computed tomography
PSI: Patient-specific Instruments
ROM: Range of motion
