# Correction to: Improving accuracy of opening-wedge osteotomies of distal radius using a patient-specific ramp-guide technique

**DOI:** 10.1186/s12891-018-2315-0

**Published:** 2018-11-19

**Authors:** Simon Roner, Fabio Carrillo, Lazaros Vlachopoulos, Andreas Schweizer, Ladislav Nagy, Philipp Fuernstahl

**Affiliations:** 1 Computer Assisted Research and Development Group, Balgrist University Hospital, University of Zurich, Forchstrasse 340, 8008 Zurich, Switzerland; 2 Department of Orthopaedics, Balgrist University Hospital, University of Zurich, Zurich, Switzerland

## Correction

Following publication of the original article [1], the author pointed out that the references were numbered incorrectly. This error was introduced during the production process. The original article has been corrected.
